# Exposure to interparental violence and justification of intimate partner violence among women in sexual unions in sub-Saharan Africa

**DOI:** 10.1186/s13690-021-00684-3

**Published:** 2021-09-09

**Authors:** Richard Gyan Aboagye, Abdul-Aziz Seidu, Bernard Yeboah-Asiamah Asare, Prince Peprah, Isaac Yeboah Addo, Bright Opoku Ahinkorah

**Affiliations:** 1 Department of Family and Community Health, School of Public Health, University of Health and Allied Sciences, Ho, Ghana; 2 Department of Population and Health, University of Cape Coast, Cape Coast, Ghana; 3 College of Public Health, Medical and Veterinary Sciences, James Cook University, Townsville, Australia; 4 Department of Estate Management, Takoradi Technical University, P.O. Box, 257, Takoradi, Ghana; 5 Curtin School of Population Health, Curtin University, Perth, Australia; 6 Institute of Applied Health Sciences, University of Aberdeen, Aberdeen, Scotland UK; 7 Social Policy Research Centre, University of New South Wales, Sydney, Australia; 8 Centre for Primary Health Care and Equity, University of New South Wales, Sydney, Australia; 9 Centre for Social Research in Health, University of New South Wales, Sydney, Australia; 10 School of Public Health, Faculty of Health, University of Technology Sydney, Sydney, Australia

**Keywords:** Interparental violence, justification of intimate partner violence, Sub-Saharan Africa

## Abstract

**Background:**

Justification of intimate partner violence (IPV) is one of the critical factors that account for the high prevalence of IPV among women. In this study, we examined the association between exposure to interparental violence and IPV justification among women in sexual unions in sub-Saharan Africa (SSA).

**Methods:**

Data for this study were obtained from the most recent Demographic and Health Surveys (DHS) of 26 countries in SSA conducted between 2010 and 2020. A total of 112,953 women in sexual unions were included in this study. A multivariable binary logistic regression analysis was carried out. The results of the regression analysis were presented using crude odds ratios (cOR) and adjusted odds ratios (aOR) with their respective 95% confidence intervals (CIs).

**Results:**

The prevalence of interparental violence in the countries considered in this study was 23.8%, with the highest (40.8%) and lowest (4.9%) in Burundi and Comoros, respectively. IPV justification was 45.8%, with the highest and lowest prevalence in Mali (80.9%) and South Africa (4.6%) respectively. Women who were exposed to interparental violence were more likely to justify IPV compared to those who were not exposed [aOR = 1.53, 95% CI = 1.47–1.59]. We found higher odds of justification of IPV among women who were exposed to interparental violence compared to those who were not exposed in all the countries, except Burkina Faso, Comoros, Gambia, and Rwanda.

**Conclusion:**

The findings call for several strategies for addressing interparental violence. These may include empowerment services targeting both men and women, formation of stronger social networks to improve women’s self-confidence, and the provision of evidence-based information and resources at the community level. These interventions should pay critical attention to young people exposed to interparental violence. Public health education and messaging should emphasise on the negative health and social implications of interparental violence and IPV.

## Background

Intimate partner violence (IPV), is described as perpetrating “physical, sexual, and emotional abuse and controlling behaviours by an intimate partner” (p1) [1]. When the violence occurs between parents, it can be termed as interparental violence [2]. Though men can also become victims of IPV, women are indicated to be the common victims of IPV around the world [1], which is seen to be more likely used by male partners as means to control [3]. Globally, it is estimated that 26% [uncertainty interval (UI) 22–30%] of women aged 15 years and over and 27% (UI 23–31%) of those aged 15–49 years have ever suffered physical and/or sexual violence by an intimate partner [4]. The prevalence of IPV in sub-Saharan Africa (SSA) (33%) is estimated to be one of the highest around the world compared to countries in Europe (16–23%) as well as Central, Eastern and South-Eastern Asia (18–21%) [4].

IPV has become a global public health problem [1]; indicated to have several dire health consequences in women including mental health problems such as depression and alcohol use disorders, sexual and reproductive health issues such as babies with low birth weights and increased risk of sexually transmitted infections (STIs) including Human Immunodeficiency Virus (HIV), injuries, and death [5]. For instance, a study indicate that IPV can increase risk of STIs and HIV by limiting a victim’s ability to negotiate safer sex [6] due to fear of further violence. It is also regarded as an abuse of human rights [7], and a social problem that has negative impact on economic empowerment, especially among women victims [8].

Several factors have been linked to the perpetration of IPV. The factors comprise socio-demographic characteristics including younger age [9], educational level [10–12], marital status [9], employment status [12], living in rural and underprivileged areas [10, 12], economic status [9, 10, 13, 14], and household decision making [14]; behavioural factors, including alcohol use [11, 12, 15, 16], smoking [9] and men who engage in fighting with other men [15]; and mental health problems [9].

Among these factors, several studies have found exposure to interparental violence [11, 13, 16, 17], and attitude toward violence (acceptance or justification of violence) [18–21] as critical predictors of perpetration of IPV and/or victimization. Exposures or experiences whether direct or distal are indicated to be predictors of behaviours [22], which have been explained by the Social Learning Theory [23]. The theory stipulates that learning of new or intensifying behaviors could be done through modeling or observing others. Thus, studies have explained that violence is a learned phenomenon that could be passed on from one generation to another [13, 17] and have suggested IPV behaviors could be acquired from childhood through modeling as they are being exposed. Gender norms that normalise and rationalise gender disparity and violence which are usually learned during childhood and significantly formed in adolescence through exposures have also been indicated to facilitate violence and victimisation [24]. Studies have found that exposure to IPV is a strong predictor of the justification of IPV [17, 19, 25]*.* Islam et al. [17] reported that men in Bangladesh who were exposed to IPV were more likely to endorse attitudes that approve IPV. Similarly, Uthman et al. [19] in Nigeria also found that women who were exposed to IPV were more likely to have acceptance attitudes toward IPV, and having tolerant attitudes was associated with experiencing physical, sexual, and emotional violence by their partners.

High levels of justification/acceptance of IPV have been identified in both male and female couples across several countries in SSA [18, 25, 26], with women identified to be more likely to justify IPV [18, 26, 27]. Studies have examined the underlying factors associated with the justification of IPV in SSA [18, 25–28]. However, most of these studies were limited to socio-demographic characteristics and/or country specific analysis [25–28] and have focused less on the effect of exposure of IPV on the justification of IPV among women in SSA [19]. Understanding the magnitude and factors associated with justification of IPV, a critical predictor of IPV, is a fundamental requirement for developing effective interventions for addressing IPV against women in societies. Using the nationally representative surveys of women aged 15–49 years from 26 sub-Saharan African countries, we examined the influence of exposure to interparental violence on women’s justification of IPV in SSA.

## Methods

### Data source and study design

Data for this study were obtained from the Demographic and Health Surveys (DHS) of 26 countries in SSA conducted between 2010 and 2020. We utilised the datasets from the individual recode files. DHS is a cross-sectional nationally representative survey that gathers data on several health indicators including domestic violence in over 85 low- and middle-income countries. DHS is mostly carried out every 5 years [29]. However, the period can be longer based on certain conditions that exist in certain countries. The DHS employs a two-stage cluster sampling technique in sampling the respondents. The first stage involves the selection of clusters usually called enumeration areas (EAs), followed by the selection of households for the survey. A detailed sampling technique and data collection procedure have been highlighted in a previous study [30]. In this study, countries were considered if they had information on the DHS domestic violence modules and had available datasets obtained between 2010 and 2020. Also, only countries with complete cases of variables of interest were included in the final analysis. A total of 112,953 women in sexual unions were included in the study. Table 1 shows the countries that were included in this study. We relied on the Strengthening Reporting of Observational Studies in Epidemiology (STROBE) reporting guidelines in drafting this paper [31]. Because of the sampling technique, which resulted in a relatively high response rate for women, the DHS is able to minimize selection bias. In addition, the survey uses standardized data gathering instruments and processes that had been well evaluated. Interviewers are also given comprehensive training to ensure that trustworthy data were collected.

**Table 1:** Description of sample

S/N, Country	Year of survey	Weighted N	Weighted %
1. Angola	2015–16	5967	5.3
2. Burkina Faso	2010	9598	8.5
3. Benin	2018	3979	3.5
4. Burundi	2016–17	6584	5.8
5. DR Congo	2013–14	4757	4.2
6. Cote d’Ivoire	2011–12	4296	3.8
7. Cameroon	2018	4128	3.7
8. Ethiopia	2016	4371	3.9
9. Gabon	2012	2917	2.6
10. Gambia	2019–20	3079	2.7
11. Kenya	2014	3627	3.2
12. Comoros	2012	2166	1.9
13. Liberia	2019–20	1668	1.5
14. Mali	2018	3179	2.8
15. Malawi	2015–16	4547	4.0
16. Nigeria	2018	8329	7.4
17. Namibia	2013	1083	1.0
18. Rwanda	2014–15	1635	1.4
19. Sierra Leone	2019	3643	3.2
20. Chad	2014–15	3064	2.7
21. Togo	2013–14	4738	4.2
22. Tanzania	2015–16	6516	5.8
23. Uganda	2016	6153	5.5
24. South Africa	2016	2062	1.8
25. Zambia	2018	5920	5.2
26. Zimbabwe	2015	4944	4.4
**All countries**		**112,953**	**100.0**

### Study variables

#### Outcome variables

The outcome variable in the present study was justification of IPV. This variable was obtained from responses to the question: “Sometimes a husband is annoyed or angered by things that his wife does. In your opinion, is a husband justified in hitting or beating his wife in the following situations?” Five situations were identified, namely going out without telling him, neglecting the children, arguing with him, refusing to have sex with him, and burning food. The responses to questions for each of these responses were coded as ‘yes’ and ‘no’. In this study, women who answered ‘yes’ to at least one of the situations for which a husband hits or beats the wife, were considered as justifying IPV while those who answered ‘no’ to all the five situations were considered as not justifying IPV [32–36].

#### Key explanatory variable

Interparental violence was the main explanatory variable in the present study. This variable was assessed using the item “whether the woman witnessed her father ever beat her mother”. The response options were 0 = No; 1 = Yes; and 8 = Don’t know. Those that responded “No” and “Don’t know” were grouped as “Not exposed” whilst those that responded “Yes” were categorised as “Exposed” to interparental violence. Similar coding was used in studies conducted in Nigeria [37] and Bangladesh [38].

### Covariates

Eleven variables were considered as covariates in this study. These variables consisted of nine individual level variables (maternal age [years], maternal educational level, marital status, maternal current working status, exposure to radio, exposure to television, exposure to newspapers/magazines, partner’s age [years], partner’s educational level) and two household level variables (wealth index and place of residence). The selection of the covariates was based on their significant associations with justification of IPV [32–36] as well as their availability in the DHS datasets. During the recoding, we maintained the existing coding in the DHS for the educational levels of the respondents and their partners (no education/primary/secondary/higher), maternal current working status (yes/no), wealth index (poorest/poorer/middle/richer/richest), and place of residence (urban/rural). Marital status was recoded as “married” and “cohabiting”. The partner’s age (years) was recoded as “15–24”, “25–34”, “35–44”, and “45+”. With exposure to radio, exposure to television, and exposure to newspapers/magazines, the respondents who responded “not at all” to reading newspapers, listening to radio, and watching television were categorised as “Not exposed [No]” whilst those who responded “less than once a week” “at least once a week” and “almost every day” were grouped as “Exposed [Yes]’.

### Statistical analyses

All analyses were performed using Stata version 16.0 (Stata Corporation, College Station, TX, USA). The prevalence of interparental violence and IPV justification were derived from descriptive statistics using percentages, with their respective 95% confidence intervals. Pearson chi-square test of independence was used to determine the relationship between exposure to interparental violence, the covariates, and IPV justification. After this, a multicollinearity test was conducted using the Variance Inflation Factor (VIF). The results showed no evidence of multicollinearity among the variables studied. Finally, a multivariable binomial logistic regression was carried out to determine the association between exposure to interparental violence and justification of IPV using three models. The first model looked at the association between exposure to interparental violence and justification of IPV without any of the covariates. The second model focused on the association between exposure to interparental violence and justification of IPV while controlling for the individual-level variables. The final model measured the association between exposure to interparental violence and justification of IPV while controlling for all the covariates. The results of the regression analyses were presented using adjusted odds ratios (aOR) and their respective 95% confidence intervals (CIs). Statistical significance was set at *p* < 0.05. The women’s sample weights for the domestic violence module (d005/1,000,000) was applied to obtain unbiased estimates according to the DHS guidelines and the survey command (svy) in Stata was used to adjust for the complex sampling structure of the data in the chi-square and regression analyses.

## Results

### Prevalence of exposure to interparental violence and justification of intimate partner violence among women

The prevalence of interparental violence in the 26 countries considered in this study was 23.8%, with the highest (40.8%) and lowest (4.9%) in Burundi and Comoros, respectively. The prevalence of IPV justification was 45.8%. The highest and lowest prevalence of IPV justification were found in Mali (80.9%) and South Africa (4.6%) respectively (Table 2).

**Table 2:** Prevalence of women’s exposure to interparental violence and IPV Justification among women in SSA

S/N, Country	Exposure to interparental violence	IPV Justification
1. Angola	27.5 [25.5, 29.6]	27.7 [25.0, 30.5]
2. Burkina Faso	8.9 [7.8, 10.0]	45.5 [43.1, 47.8]
3. Benin	9.5 [8.4, 10.7]	31.8 [29.5, 34.1]
4. Burundi	40.8 [39.3, 42.4]	61.9 [59.7, 64.0]
5. DR Congo	33.3 [30.9, 35.8]	76.3 [73.5, 78.9]
6. Cote d’Ivoire	14.0 [12.4, 15.8]	50.0 [46.6, 53.3]
7. Cameroon	21.0 [18.9, 23.1]	31.2 [28.4, 34.1]
8. Ethiopia	27.6 [25.1, 30.2]	67.5 [64.5, 70.4]
9. Gabon	33.3 [30.2, 36.5]	50.6 [47.2, 53.9]
10. Gambia	9.2 [7.4, 11.3]	60.2 [56.1, 64.2]
11. Kenya	36.6 [34.6, 38.7]	43.0 [40.7, 45.3]
12. Comoros	4.9 [3.7, 6.5]	39.3 [35.6, 43.0]
13. Liberia	23.5 [20.3, 27.0]	39.9 [36.3, 43.6]
14. Mali	10.1 [8.6, 11.7]	80.9 [78.5, 83.1]
15. Malawi	26.0 [24.3, 27.8]	15.3 [13.8, 16.9]
16. Nigeria	10.3 [9.4, 11.4]	27.6 [25.5, 29.7]
17. Namibia	24.9 [21.8, 28.3]	38.3 [26.9, 34.3]
18. Rwanda	38.5 [36.1, 41.0]	38.3 [35.3, 41.4]
19. Sierra Leone	28.0 [25.9, 30.3]	53.4 [50.3, 56.4]
20. Chad	18.2 [16.1, 20.4]	72.5 [69.6, 75.3]
21. Togo	16.0 [14.5, 17.6]	30.2 [27.6, 33.0]
22. Tanzania	36.5 [34.6, 38.5]	60.9 [58.9, 62.9]
23. Uganda	35.7 [34.0, 37.3]	49.8 [47.9, 51.7]
24. South Africa	15.7 [13.3, 18.4]	4.6 [3.5, 6.0]
25. Zambia	29.2 [27.2, 31.3]	48.9 [46.3, 51.5]
26. Zimbabwe	34.7 [32.8, 36.7]	37.1 [35.0, 39.2]
**All countries**	**23.8 [23.5, 24.0]**	**45.8 [45.5, 46.1]**

#### Distribution of justification intimate partner violence across exposure to interparental violence and covariates

Table 3 shows the distribution of IPV justification across exposure to interparental violence and covariates. The results showed significant disparities in IPV justification across exposure to interparental violence at *p* < 0.005. Specifically, IPV justification was higher among women who were exposed to interparental violence (53.6%) compared to those who were not exposed (43.4%). With the covariates, maternal age, maternal educational level, marital status, exposure to radio, exposure to television, exposure to newspaper/magazine, partner’s age, partner’s educational level, wealth index, and place of residence had significant associations with physical, emotional, and sexual violence at p < 0.005.

**Table 3:** Bivariate analysis of exposure to interparental violence and intimate partner violence justification among women in sub-Saharan Africa

Variable	Weighted N	Weighted %	IPV Justification	
			Yes (%)	***p***-value
**Exposure to interparental violence**				< 0.001
No	86,119	76.2	43.4	
Yes	26,834	23.8	53.6	
**Maternal age**				< 0.001
15–19	7047	6.2	55.1	
20–24	20,611	18.4	49.1	
25–29	25,924	22.9	45.4	
30–34	22,551	20.0	43.7	
35–39	17,197	15.2	43.8	
40–44	11,450	10.1	43.5	
45–49	8172	7.2	43.9	
**Maternal educational level**				< 0.001
No education	42,085	37.3	53.8	
Primary	38,024	33.7	49.0	
Secondary	27,983	24.8	34.9	
Higher	4861	4.3	14.8	
**Marital status**				< 0.001
Married	88,706	78.5	46.4	
Cohabiting	24,247	21.5	43.6	
**Current working status**				0.075
No	36,356	32.2	46.5	
Yes	76,597	67.8	45.5	
**Exposure to radio**				< 0.001
No	48,302	42.8	49.4	
Yes	64,651	57.2	43.2	
**Exposure to television**				< 0.001
No	68,559	60.7	51.6	
Yes	44,394	39.3	36.9	
**Exposure to newspaper/magazine**				< 0.001
No	92,892	82.2	48.6	
Yes	20,061	17.8	32.9	
**Partner’s age**				< 0.001
15–24	6778	6.0	50.9	
25–34	37,924	33.6	46.3	
35–44	37,893	33.5	44.4	
45+	30,358	26.9	45.9	
**Partner’s educational level**				< 0.001
No education	34,274	30.3	54.3	
Primary	34,569	30.6	49.3	
Secondary	35,057	31.1	39.3	
Higher	9053	8.0	25.7	
**Wealth index**				< 0.001
Poorest	22,757	20.2	54.3	
Poorer	23,134	20.5	51.9	
Middle	22,620	20.0	48.8	
Richer	22,726	20.1	42.5	
Richest	21,716	19.2	30.7	
**Place of residence**				< 0.001
Urban	39,424	34.9	34.5	
Rural	73,529	65.1	51.9	

*P*-values are from Chi-square Test

### Association between exposure to inter-parental violence and justification of intimate partner violence among women in sub-Saharan Africa

Model III of Table 4 shows the results of the association between interparental violence and IPV justification among women in SSA. We found that women who were exposed to IV were more likely to justify IPV compared to those who were not exposed [aOR = 1.53, 95% CI = 1.47–1.59][aOR = 1.53, 95% CI = 1.47–1.59]. We found higher odds of IPV justification among women who were exposed to interparental violence compared to those who were not exposed in all the 26 countries except Burkina Faso, Comoros, Gambia, and Rwanda (Table 5, Model II). We found higher odds of IPV justification of among women who were exposed to interparental violence compared to those who were not exposed in all the 26 countries except Burkina Faso, Comoros, Gambia, and Rwanda (Table 5, Model II) In terms of the covariates, maternal age [years], maternal educational level, marital status, maternal current working status, exposure to television, exposure to newspapers/magazines, partner’s age [years], partner’s educational level, wealth index and place of residence showed significant associations with justification of IPV (Table 4, Model III).

**Table 4:** Multivariable regression analysis of women’s exposure to interparental violence and IPV Justification in sub-Saharan Africa

Variables	Model I cOR [95% CI]	Model II aOR [95% CI]	Model III aOR [95% CI]
**Exposure to interparental violence**			
No	1 [1.00,1.00]	1 [1.00,1.00]	1 [1.00,1.00]
Yes	1.51^***^ [1.45,1.57]	1.56^***^ [1.50,1.62]	1.53^***^ [1.47,1.59]
**Maternal age**			
15–19		1 [1.00,1.00]	1 [1.00,1.00]
20–24		0.84^***^ [0.78,0.90]	0.84^***^ [0.78,0.90]
25–29		0.72^***^ [0.66,0.77]	0.72^***^[0.67,0.78]
30–34		0.64^***^ [0.59,0.70]	0.65^***^ [0.60,0.70]
35–39		0.60^***^ [0.55,0.66]	0.61^***^ [0.56,0.67]
40–44		0.56^***^ [0.51,0.62]	0.57^***^ [0.52,0.63]
45–49		0.55^***^ [0.49,0.60]	0.55^***^ [0.49,0.61]
**Maternal educational level**			
No education		1 [1.00,1.00]	1 [1.00,1.00]
Primary		0.88^***^ [0.85,0.92]	0.89^***^ [0.85,0.93]
Secondary		0.61^***^ [0.57,0.64]	0.65^***^ [0.61,0.69]
Higher		0.26^***^ [0.22,0.29]	0.29^***^ [0.25,0.33]
**Marital status**			
Married		1 [1.00,1.00]	1 [1.00,1.00]
Cohabiting		0.93^**^ [0.88,0.98]	0.94^*^ [0.89,0.99]
**Exposure to television**			
No		1 [1.00,1.00]	1 [1.00,1.00]
Yes		0.78^***^ [0.74,0.82]	0.91^***^ [0.86,0.95]
**Exposure to radio**			
No		1 [1.00,1.00]	1 [1.00,1.00]
Yes		1.01 [0.97,1.05]	1.01 [0.97,1.05]
**Exposure to newspaper/magazine**			
No		1 [1.00,1.00]	1 [1.00,1.00]
Yes		0.90^***^ [0.85,0.95]	0.91^***^ [0.87,0.96]
**Partner’s age**			
15–24		1 [1.00,1.00]	1 [1.00,1.00]
25–34		1.06 [0.99,1.15]	1.09^*^ [1.01,1.17]
35–44		1.13^**^ [1.03,1.23]	1.16^***^ [1.07,1.27]
45+		1.18^***^ [1.07,1.29]	1.22^***^ [1.11,1.34]
**Partner’s educational level**			
No education		1 [1.00,1.00]	1 [1.00,1.00]
Primary		0.88^***^ [0.84,0.93]	0.89^***^ [0.85,0.94]
Secondary		0.77^***^ [0.73,0.81]	0.81^***^ [0.77,0.86]
Higher		0.67^***^ [0.61,0.73]	0.74^***^ [0.68,0.82]
**Wealth index**			
Poorest			1 [1.00,1.00]
Poorer			0.98 [0.93,1.03]
Middle			0.97 [0.91,1.02]
Richer			0.90^**^ [0.85,0.96]
Richest			0.79^***^ [0.73,0.86]
**Place of residence**			
Urban			1 [1.00,1.00]
Rural			1.30^***^ [1.22,1.39]

NB: Model III adjusted for maternal age, maternal educational level, marital status, exposure to television, exposure to radio, exposure to newspaper/magazine, partner’s age, partner’s educational level, wealth index and place of residence

Exponentiated coefficients; 95% confidence intervals in brackets, ^*^ *p* < 0.05, ^**^ *p* < 0.01, ^***^ *p* < 0.001, *cOR* Crude odds ratio, *aOR* Adjusted odds ratio, *CI* Confidence interval

**Table 5:** Regression analysis of women’s exposure to interparental violence and IPV Justification among women in SSA by country

S/N, Country	Model I cOR [95% CI]	Model II aOR [95% CI]
1. Angola	1.58*** [1.40, 1.78]	1.74*** [1.53, 1.97]
2. Burkina Faso	0.92 [0.80, 1.06]	0.94 [0.82, 1.08]
3. Benin	1.70*** [1.37, 2.10]	1.67*** [1.34, 2.09]
4. Burundi	1.26*** [1.13, 1.39]	1.18** [1.06, 1.31]
5. DR Congo	1.64*** [1.42, 1.88]	1.60*** [1.38, 1.84]
6. Cote d’Ivoire	1.27** [1.06, 1.52]	1.26* [1.05, 1.51]
7. Cameroon	1.27** [1.08, 1.50]	1.34** [1.13, 1.59]
8. Ethiopia	1.34*** [1.16, 1.56]	1.36*** [1.16, 1.60]
9. Gabon	1.74*** [1.51, 2.01]	1.62*** [1.40, 1.89]
10. Gambia	0.90 [0.71, 1.14]	0.76* [0.59, 0.98]
11. Kenya	1.53*** [1.34, 1.75]	1.43*** [1.25, 1.64]
12. Comoros	1.02 [0.69, 1.53]	0.99 [0.66, 1.49]
13. Liberia	1.60*** [1.30, 2.00]	1.47*** [1.20, 1.81]
14. Mali	1.63** [1.17, 2.26]	1.44* [1.02, 2.02]
15. Malawi	1.25* [1.04, 1.49]	1.28** [1.07, 1.53]
16. Nigeria	1.27** [1.10, 1.47]	1.39*** [1.19, 1.62]
17. Namibia	1.95*** [1.49, 2.56]	1.74*** [1.29, 2.34]
18. Rwanda	1.17 [0.95, 1.44]	1.13 [0.92, 1.41]
19. Sierra Leone	1.24** [1.07, 1.44]	1.24** [1.07, 1.44]
20. Chad	2.42*** [1.88, 3.12]	1.83*** [1.40, 2.38]
21. Togo	1.98*** [1.71, 2.30]	1.69*** [1.45, 1.98]
22. Tanzania	1.90*** [1.70, 2.12]	1.68*** [1.50, 1.88]
23. Uganda	2.29*** [2.06, 2.55]	2.05*** [1.84, 2.29]
24. South Africa	2.24*** [1.43, 3.50]	2.16** [1.36, 3.43]
25. Zambia	1.79*** [1.60, 2.01]	1.69*** [1.50, 1.91]
26. Zimbabwe	1.49*** [1.32, 1.68]	1.44*** [1.26, 1.64]

Exponentiated coefficients; 95% confidence intervals in brackets, *p < 0.05, **p < 0.01, ***p < 0.001, *cOR* Crude odds ratio, *aOR* Adjusted odds ratio, *CI* Confidence interval

## Discussion

In the most recent fact sheets on domestic violence released by the WHO [4], almost one third (27%) of women aged 15–49 years have ever experienced physical and/or sexual violence by their intimate partners worldwide. Thus, the present study examined the association between exposure to interparental violence and justification of IPV among women in sexual unions in 26 SSA countries. This association is worth investigating because increased justification of IPV by women in unions has several implications. For instance, acceptance of IPV reduces the likelihood of women reporting and seeking help, and increases the risk of women experiencing more episodes of spousal or partner abuses in the future [17]. The results of this multi-country representative analysis demonstrated that nearly 24% of women in sexual unions have been exposed to interparental violence and almost 46% of these women endorsed attitudes justifying IPV. The prevalence of exposure to interparental violence in our study is comparable to rates reported in developing countries, for example, among men (27%) in Bangladesh [38, 39], but higher than the reported situation in many developed countries (e.g., France (21%) [40].

The differences in the rates could be attributed to the variations in the study procedures, methodologies, samples, and study settings. Interparental violence justification of IPV was higher among women who were exposed to interparental violence (53.6%) compared to those who were not exposed (43.4%), which was statistically significant. Similar findings have been reported in previous studies in developing countries. For instance, using the 2015–2016 round of the National Family and Health Survey in India, women who experienced parental violence in their childhood reported higher IPV [41]. Another recent study conducted among 847 college students aged between 18 to 25 years found that more students who were exposed to interparental violence experienced violence in their intimate relationships [42] compared to those who had not experienced any form of interparental violence. The consistency in findings suggests that interparental violence may play an important role in intimate relationships [42]. This indicates that further attention should be paid to children exposed to interparental violence.

Consistent with our main argument, we found that having been exposed to interparental violence increased the likelihood of justifying IPV among women in unions in SSA. Further, higher odds of justifying IPV among women who were exposed to interparental violence compared to those who were not exposed in all the 26 countries except Burkina Faso, Comoros, Gambia, and Rwanda were found. The take-home evidence that being exposed to interparental violence increases the likelihood of justifying IPV mirrors findings from previous studies in Uganda [43, 44], Ethiopia [43], Bangladesh [36, 37], Philippines [45], Haiti [46], India [41] and Spain [42], where those who were exposed to interparental violence supported spousal or partner abuse compared with those who have not experienced such violence. Our findings, therefore, reinforce this literature and suggest this link is similarly prominent in SSA.

Plausible explanations for this link, specifically, pathways through which being exposed to interparental violence could be related to the increased likelihood of justifying intimate partner violence by women have been offered by previous studies (see [38, 46, 47]). For instance, Gage [46] offered the explanation that women exposed to interparental or intra-family violence may form mental representations of relationships that increase their vulnerability to violence exposure in intimate relationships. As a consequence of witnessing father and mother hitting each other, women may construct attachment models along dominance-subordination and victim-victimizer dimensions [48, 49]. Thus, women may select partners and situations that are consistent with their understanding of what relationships are about, who they are in relationships, and what to expect from a relationship partner [46]. Our finding is therefore consistent with the multi-generational effect of violence literature [50]. Another possible explanation is that women who were exposed to interparental violence may perceive intimate partner violence as a normal part of intimate relationships especially in the SSA settings where intimate relationships are built and prescribed by cultural beliefs and conceptualizations. This explains Kwagala et al. [43] observation in Uganda that experiences of interparental violence become part of socialization that nurtures attitudes that accept or justify IPV. Thus, domestic violence may form part of a lifelong continuum, beginning with childhood violence exposure in the family of origin and continuing with violence in intimate relationships and families formed in adulthood [46]. From the analysis, we do not know whether this intergenerational or multigenerational effect of domestic violence characterized perpetrators as well. Notwithstanding, the results strongly suggest the need for early identification of intimate partner violence and intervention for the entire family to reduce the likelihood that abused women’s children experience abuse themselves in adulthood as either victims or perpetrators.

Aside from the major finding of the study, other findings of the analysis are worthy of highlight. Socio-demographic factors namely age, education, marital status, exposure to social media, wealth index, and place of residence were associated with IPV justification. Having a tertiary level of education, cohabiting, being exposed to television and newspaper/magazines reading, and falling within the richest wealth quintile were mitigating factors for justifying IPV among women, whereas living in a rural area and increasing partner’s age were risk factors for IPV justification among women. This finding highlights the importance of analysis of the context-specific socio-demographic and economic factors when addressing intimate partner violence. The finding also suggests that women’s autonomy, empowerment, and economic independence programmes and interventions may help address their socio-culturally and demographically framed IPV justification in SSA.

### Strengths and limitations

The study has presented evidence that reinforces the significance of exposure to interparental violence in the justification of IPV among women in sub-Saharan Africa, which could have essential implications for health policy and interventions on IPV in the region. The study also relied on relatively large data from nationally representative samples from several countries and as such enhances the accuracy and generalisation of the findings.

However, the findings of this study are limited to some extent. Firstly, the study relied on cross-sectional data and as such causal interpretations of the findings are limited. Secondly, the study relied on data collected through self-reporting which could not be independently verified, and as such the prevalence of IPV and exposure to interparental violence could be under-or overestimated. Moreover, the data used for this study was limited to only women and that is consistent with the common belief that women are the usual victims of intimate partner violence. While this common belief remains contestable in the future, this present evidence-based study provides timely and important information that can be used to address the current victimization of women in intimate partner violence within the SSA region.

### Policy and public health implications

Our finding that being exposed to interparental violence increases the likelihood of justifying IPV has both policy and public health implications. The study finding calls for either the consolidation of existing policies and progammes or the creation of new policies and programmes that address interparental violence and IPV in SSA. The complexity of interparental violence and its association with IPV justification as well as demographic and economic factors suggest that single policies and programmes are unlikely to produce desirable and long-standing change and outcomes thus, comprehensive and multifaceted approaches and strategies are required. Strategies for addressing interparental violence may include empowerment services targeting both men and women, greater social networks and self-confidence of women, provision of information and resources at the community and societal level. These intervention models should pay critical attention to young people who have been exposed to interparental violence. Public health education and messaging about the negative health and social implications of interparental violence and intimate partner should be enhanced in most communities especially the rural areas of SSA. Strategies for addressing interparental violence and intimate partner violence justification face particular obstacles in SSA contexts due to issues such as poverty, little or no access to appropriate domestic abuse information and services, illiteracy, inadequate legal redress for victims of violence and socio-cultural norms, values, and practices. However, strategies and services that are sensitive to the cultural context of individuals involved in interparental violence and intimate partner violence justification could be the appropriate ones to foster long-standing change and outcomes.

## Conclusion

In this multi-country analysis of 26 nationally representative surveys, a statistically significant association between exposure to interparental violence and IPV was found; demographic and economic factors such as wealth index, education, exposure to mass media, among others also play a significant role in women’s IPV experience. In such a context, intervention models to address interparental violence are needed especially to pay attention to young people who are exposed to such violence to prevent their future IPV justification. We also recommend that interventions and policies should consider social and cultural contexts. Importantly, from our analysis, we do not know whether this intergenerational or multigenerational effect of domestic violence characterized perpetrators as well. It would therefore be interesting for future studies to assess whether the intergenerational or multigenerational effects of domestic violence characterized perpetrators in SSA. Moreover, qualitative inquiry into the socio-cultural and economic issues surrounding interparental violence and IPV justification would be important.

## Data Availability

Dataset for this study is freely available for download at: http://dhsprogram.com/data/available-datasets.cf.

## Abbreviations

AIC: Akaike’s information criterion
aOR: adjusted Odds Ratio
CI: Confidence Interval
DHS: Demographic and Health Surveys
EA: Enumeration areas
ICC: Intra-cluster correlation
IPV: Intimate partner violence
IV: Interparental violence
SSA: Sub-Saharan African
STIs: Sexually transmitted infections
VIF: Variance inflation factor
WHO: World Health Organisation
